# Transcriptomic analysis revealed that oar-miR-370-3p was involved in the regulation of wool fineness in Ordos fine-wool sheep

**DOI:** 10.3389/fvets.2025.1662962

**Published:** 2025-10-24

**Authors:** Cuiling Wu, Gvlnigar Amar, Asma Anwar, Wenna Liu, Sen Tang, Shengchao Ma, Xuefeng Fu

**Affiliations:** ^1^Key Laboratory of Special Environment Biodiversity Application and Regulation in Xinjiang, International Center for the Collaborative Management of Cross-border Pest in Central Asia, College of Life Sciences, Xinjiang Normal University, Ürümqi, Xinjiang, China; ^2^Key Laboratory of Genetic Breeding and Reproduction of Herbivorous Livestock, Ministry of Agriculture and Rural Affairs, Xinjiang Key Laboratory of Animal Biotechnology, Institute of Biotechnology, Xinjiang Academy of Animal Sciences, Ürümqi, Xinjiang, China

**Keywords:** Ordos fine-wool sheep, wool, fiber diameter, oar-miR-370-3p, fibroblast

## Abstract

MicroRNA (miRNA) is a prominent topic in biological research, as it plays a crucial role in regulating various physiological processes. Identifying miRNAs associated with fiber traits enhances our understanding of the complex biological mechanisms involved in hair follicle development and provides potential targets for improving fine-wool sheep breeds. This study focused on 20 Ordos fine-wool sheep and identified miRNA expression profiles in skin tissues through transcriptome sequencing. Key miRNAs related to the diameter of wool fibers were preliminarily screened through cell verification experiments. The results revealed 26 differentially expressed (DE) miRNAs in tissues with varying wool fineness, with 12 DE miRNAs being upregulated and 14 DE miRNAs being downregulated. All DE miRNAs predicted 2,844 target genes. Integrating previous mRNA sequencing data, oar-miR-370-3p has 23 differentially expressed target genes. Following the overexpression of miR-370-3p in fibroblasts, the expression levels of the predicted target genes *ZDHHC15*, *JUNB*, *TGFBI*, and *IFI6* were upregulated compared to the control group, resulting in increased cell activity. These results provide new insights into the molecular mechanisms regulating the diameter traits of sheep wool fibers and offer important resources for precise sheep breeding research.

## 1 Introduction

Sheep play a vital role in the livestock economy, particularly in arid, semi-arid, and hilly regions. Wool, one of the important livestock products derived from sheep, serves as an essential raw material for the textile industry. It possesses several advantages, including excellent elasticity, strong moisture absorption, and effective heat retention. Due to these characteristics, sheep are considered ideal models for studying the genetic mechanisms underlying fiber mass. Furthermore, understanding the genetic and biological processes that govern wool characteristics is crucial for enhancing the economic viability of the sheep industry. The Ordos fine-wool sheep is a characteristic livestock breed in Ordos City, Inner Mongolia Autonomous Region. Through years of selective breeding and development, it has become a vital pillar of the local animal husbandry sector. This breed has enhanced the quality of life for local farmers and herdsmen, ensuring stable income growth and making a significant contribution to the region’s economic development (1, 2). The fineness of wool, a key indicator for assessing wool quality, is essential for the economic value and market competitiveness of fine-wool sheep. In the woolen textile industry, wool fineness is directly linked to the quality and grade of the finished product (3).

It is well known that miRNAs control the expression of mRNA through base complementary pairing and can target non-coding RNAs, including long non-coding RNAs (lncRNAs) and circular RNAs (circRNAs). Due to their complex regulatory mechanisms, miRNAs have become an interesting field in the study of animal growth and development, physiological activities and biological functions. Research across multiple animal models has demonstrated that miRNAs play an important role in hair formation. In human studies, miR-221 has been shown to significantly inhibit hair growth and the proliferation of dermal papillary cells and dermal sheath cells in patients with androgenetic alopecia (4).

In the study of Aohan fine-wool sheep, it was found that the miR-21 is associated with hair follicle development (5). Research on both normal and hairless pigs indicates that miR-29a-5p inhibits the proliferation of hair placode precursor cells by suppressing the expression of EDAR within the EDA/EDAR signaling pathway, while lncRNA627.1 restores EDAR expression (6). Our previous research found that chi-miR-105a (7) in Tibetan cashmere goats is linked to the diameter of cashmere fibers, and chi-miR-877-3p (8) in Jiangnan cashmere goats is associated with the periodic development of hair follicles, which may influence cashmere yield. Although multiple studies have demonstrated a correlation between miRNA and hair formation, the comprehensive molecular mechanism behind this relationship is still not fully understood.

At present, significant progress has been made in identifying the key genes of wool traits, but the comprehensive gene database involving hair follicle development and wool growth is still incomplete. Further research is necessary to identify and characterize these genes to enhance our understanding of the genetic basis of wool production. In this study, Ordos fine-wool sheep were used as experimental animals. Based on transcriptome sequencing, miRNAs related to regulating the diameter traits of wool fibers were explored. The research results are crucial for clarifying how non-coding RNAs affect fiber properties. This knowledge provides experimental reference for precise breeding strategies to improve the quality of wool.

## 2 Materials and methods

### 2.1 Sample collection

The 12-month-old ewe of the Ordos fine-wool sheep was taken as the research object. The source of the animals is Wushen Banner Bayinsurike Agriculture and Animal Husbandry Development Co., LTD. All ewes were raised under the same conditions and were in good health. The average fiber diameter (MFD) of all wool samples was detected by using the OFDA2000 measuring instrument. Individuals with an average wool fiber diameter of 15–17 μm were selected as the fine (Fe) group (*n* = 10), and individuals within the range of an average wool fiber diameter of 18–21 μm were selected as the coarse (Ce) group (*n* = 10). The skin tissues of the left scapular region of the experimental animals were collected. After being washed with DEPC, the skin tissues were cut into pieces of approximately 2 cm^2^ and packaged separately, and then stored in liquid nitrogen.

### 2.2 Sequencing and quality control

Nucleic acid extraction was performed from the skin tissues of 20 Ordos fine-wool sheep using the miRNeasy Mini Kit (Qiagen, Hilden, Germany). The concentration and purity of the RNA were detected according to the previous research methods (8). For each sample, 20 small RNA libraries were constructed using 3 μg of RNA. The quality of these small RNA libraries was evaluated using a DNA high-sensitivity chip on the Agilent Bioanalyzer 2,100 system (Agilent, CA, United States). The Illumina Hiseq 2,500 sequencer (Illumina, San Diego, United States) was used to sequence 20 small RNA libraries and generate 50 bp single-ended reads. Clean data is obtained from the original data following quality control procedures. Concurrently, the Q20, Q30, and GC content of the data were calculated. The small RNA tags were mismatched to the reference sequence (ARS-UI_Ramb_v2.0, GCF_016772045.1) using Bowtie software. Additionally, the small RNA tags are mapped to the RepeatMasker and Rfam databases to remove the tags from protein-coding genes, repeat sequences, rRNA, tRNA, snRNA, and snoRNA.

### 2.3 Analysis of miRNAs expression levels

Novel miRNA predictions were made based on the hairpin structure characteristics of miRNA precursors. The expression levels of miRNAs were quantified and normalized using TPM algorithm. To identify the miRNAs with statistically significant differential expression between the Fe group and the Ce group of Ordos fine-wool sheep, the DESeq R software package had a *p* value threshold of ≤0.05. Target genes of the miRNAs were predicted using miRanda. Functional annotations of the predicted target genes of DE miRNAs included Gene Ontology (GO) and Kyoto Encyclopedia of Genes and Genomes (KEGG) analyses.

### 2.4 Functional verification of miRNA

The mimics and negative control (NC) were synthesized based on the oar-miR-370-3p sequence. Cells transfected with the mimics were used as the experimental group, while the NC was used as the control group. The cells were inoculated in 6-well plates and transfected according to the instructions provided for Lipofectamine 3,000 (Invitrogen, CA, United States), with 12 replicates established in each group. After transfection for 6 h, the cells were washed three times with phosphate-buffered saline (PBS), and then complete medium was added for continued culture for an additional 24 h.

The total RNA from the transfected cells was extracted using TriZol reagent (Invitrogen, CA, United States). The RNA concentration was measured using the Thermo NANODROP-2000. RNA was reverse transcribed into cDNA using the miRcute Plus miRNA First-Strand cDNA Kit and FastKing RT Kit (Tiangen, Beijing, China). The Quantitative PCR (qPCR) analysis on the LineGene 9,600 quantitative PCR detection system (Bioer Technology, Hangzhou, China) was performed using the miRcute Plus miRNA qPCR Kit and Talent qPCR PreMix (Tiangen, Beijing, China). Among them, U6 was used as the internal reference gene for miRNA quantification, while *β*-actin served as the internal reference gene for mRNA quantification. The predictive target genes of oar-miR-370-3p included *ZDHHC15*, *JUNB*, *TGFB*I, *IFI6*, *CDHR5*, and *PPT2*, and their expression levels were assessed across different transfection groups. Each experimental sample experiment was repeated three times. The relative expression levels of miRNA and mRNA were calculated using the 2^-ΔΔCt method. The primer sequences are provided in Table 1.

**Table 1:** Primer information.

Gene	Forward primer sequence(5′-3′)	Reverse primer sequence(5′-3′)
oar-miR-370-3p	GCCTGCTGGGGTGGAACCTGGTCT	–
U6	ACGGACAGGATTGACAGATT	–
*β-actin*	CTTCCAGCCTTCCATCCCGG	GCCAGGGCAGTGTTCTCTTT
*ZDHHC15*	GCCTGGTGTTCGCTCTAAGT	TGCTGACAAGCCAACAATGG
*JUNB*	CGACGAATCATACGGAACAGC	GTGTCCGAACCCTGGCTAGA
*TGFBI*	TACGGGACCCTGTTCTCCAT	AGCATGCTGAAGCGATTGTC
*IFI6*	CCTGTGCTACCTGCTACTCT	CAGAAGCTCGAGTCGCTGT
*CDHR5*	GTCTTCTACGCCGAGGTCAAG	GAGTCTGGTAGTTCCTGCCG
*PPT2*	CCCAGCGGCGGGAGT	TATGACCGGCTTGTAGGCAG

### 2.5 Cell proliferation detection

Cells in the logarithmic growth phase were taken and transfected according to the miRNA mimics, NC and blank groups. The transfected cells were treated with trypsin solution, and the cells were collected with culture medium to prepare a cell suspension, which was then inoculated into 96-well plates, with 10 replicates in each group. After the cells adhered and grew for 24 h, the cells containing 10% CCK-8 reagent (Servicebio, Wuhan, China) were inoculated. A volume of 100 μL of medium was added to each well and incubated at 37 °C for 2 h. The optical density was measured at 450 nm by an microplate reader to calculate the survival rate of fibroblasts. Each experimental sample was repeated four times.

## 3 Results

### 3.1 Statistics of sequencing data

The sequencing data volume of all samples ranged from 0.55 G to 0.9 G. The percentages of Q20 and Q30 exceeded 98.86 and 96.73%, respectively, indicating the high quality of the sequencing data. The GC content ranged from 48.83 to 49.45%. The clean reads were aligned to the reference genome of sheep, with the mapped sRNA ranging from 96.87 to 97.41%. Overall, these data demonstrate that all samples exhibit good sequencing quality, a low error rate, and high reliability.

### 3.2 Differentially expressed miRNA analysis

**Figure 1:**
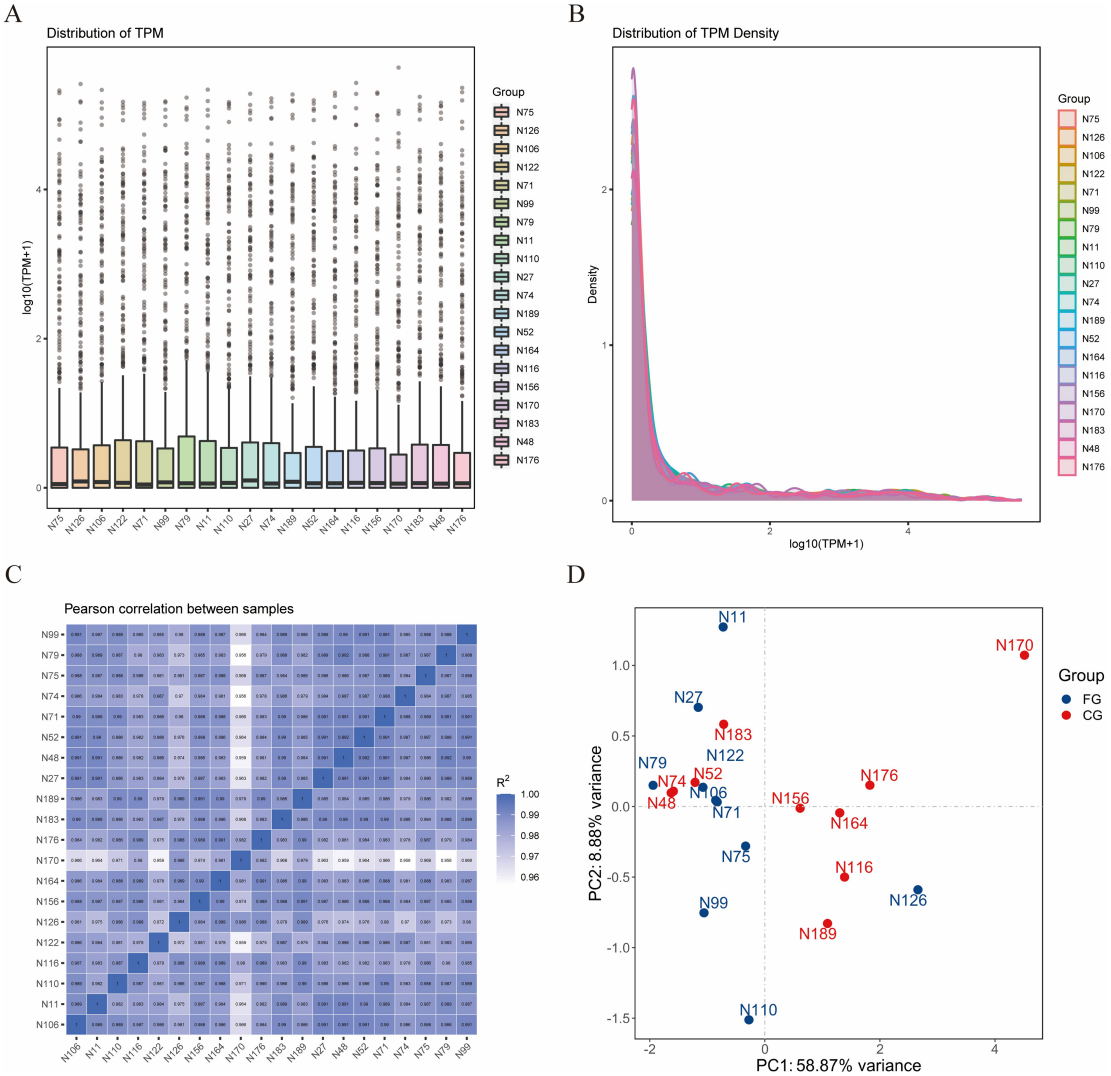
Statistical analysis of miRNA expression profiles. **(A)** Box plot of miRNA expression level. **(B)** Comparison chart of miRNA expression level density distribution. **(C)** Correlation heat maps of all samples. **(D)** PCA plots of all samples.

**Figure 2:**
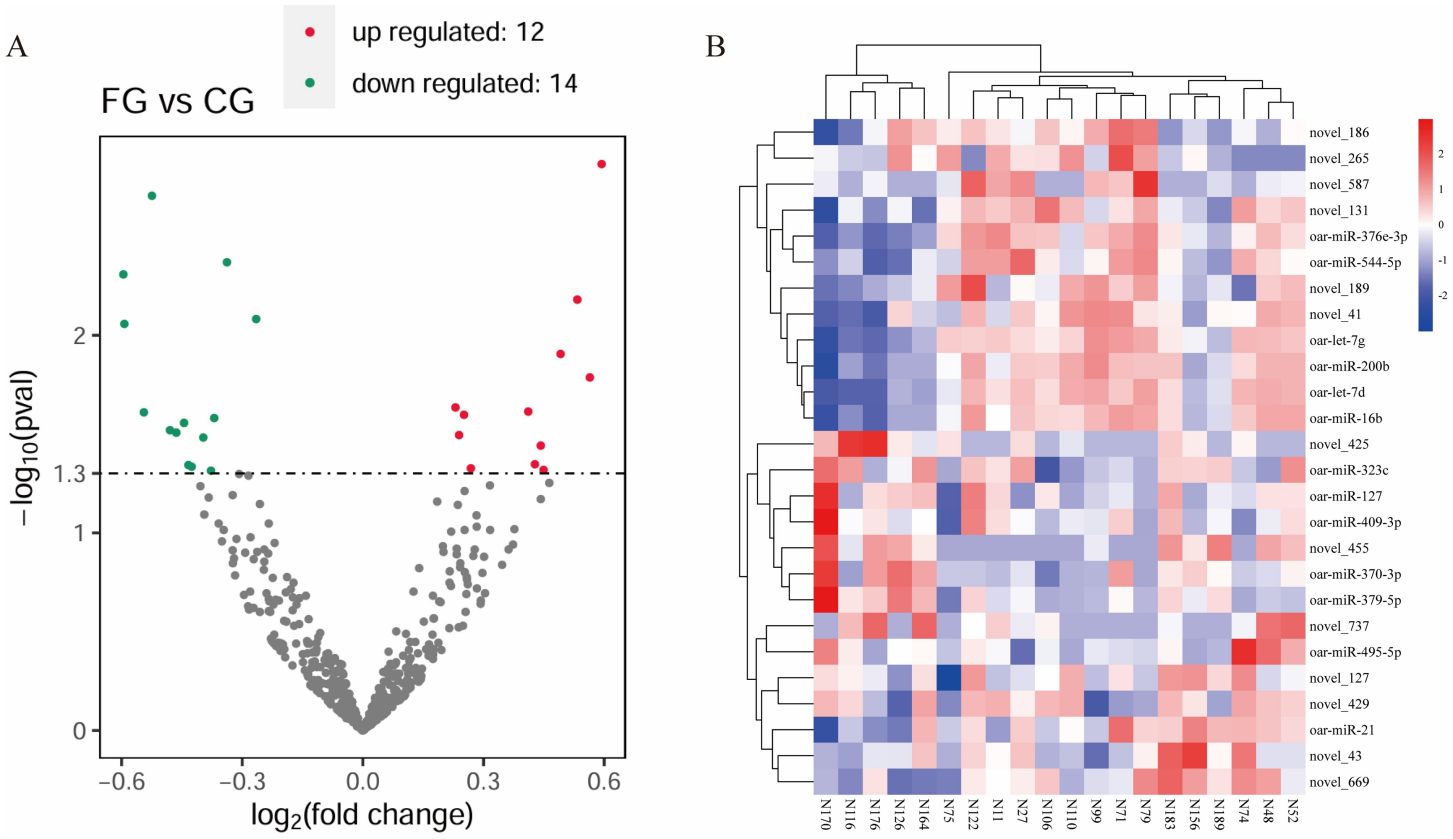
Statistical analysis of differentially expressed miRNAs. **(A)** Volcanic map of DE miRNA. Green dots indicate down-regulated miRNAs, red dots indicate up-regulated miRNAs, and black dots indicate miRNAs with no differential expression. **(B)** Heat map of DE miRNA. The different columns in the figure represent different samples, and the different rows represent different miRNAs.

The box plot and density distribution map were drawn based on the TPM values of miRNAs (Figures 1A,B). The results indicated that the gene expression levels across the 20 samples were relatively stable. By calculating the correlation of gene expression levels between each pair of samples, Figure 1C demonstrates that the correlation among all samples is relatively high. PCA analysis was performed on the 20 samples, and no obvious separation occurred between the samples in the FG group and the CG group (Figure 1D). An analysis of the miRNA expression matrix revealed that a total of 517 miRNAs were obtained, among which 368 were novel miRNAs. The top three miRNAs in terms of average expression levels were oar-miR-148a, oar-miR-26a, and oar-miR-143. The miRNA expression profiles of skin tissues in the FG group and CG group of 20 Ordos fine-wool sheep were compared. We screened out a total of 26 differentially expressed miRNAs. Compared with the CG group, the expression of 12 miRNAs was upregulated and that of 14 miRNAs was downregulated in the FG group (Figure 2). Among all the differentially expressed miRNAs, 13 are known miRNAs, among which 6 are up-regulated and 7 are down-regulated.

### 3.3 Prediction of target genes for DE miRNAs

**Figure 3:**
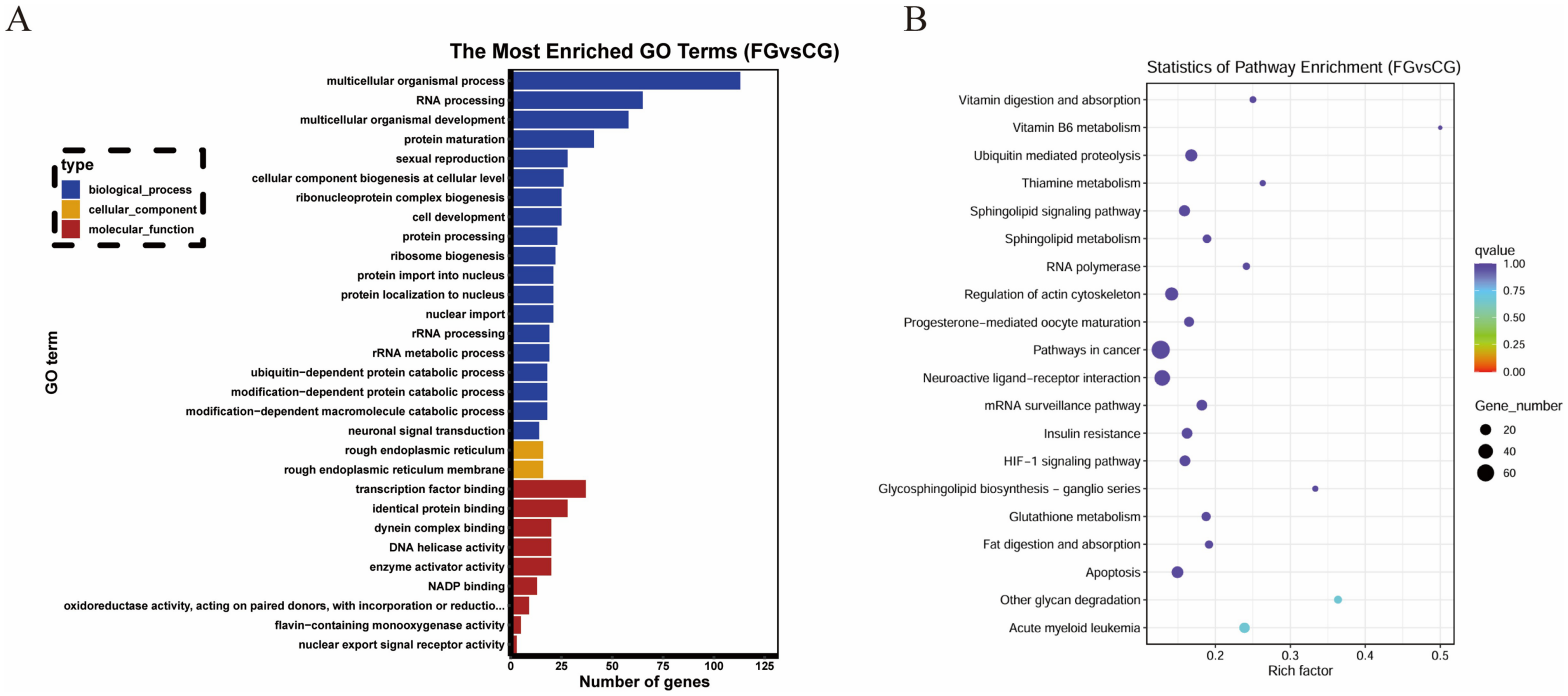
Functional enrichment analysis of DE miRNA target genes. **(A)** GO enrichment analysis. The X-axis represents the number of genes. The Y-axis represents GO terms. Bar graph the top 30 GO terms with the smallest *q* value. **(B)** KEGG enrichment analysis. The X-axis represents the enrichment factor. The Y-axis represents KEGG Pathway. Bubble graph the top 20 pathway with the smallest *q* value.

A total of 2,844 target genes were predicted for 26 DE miRNAs. According to GO analysis, the target genes were mainly enriched in GO entries such as ribonucleoprotein complex biogenesis (GO:0022613), cellular component biogenesis at cellular level (GO:0071843), and transcription factor binding (GO:0008134) (Figure 3A). Based on KEGG analysis, the target genes were predominantly enriched in signaling pathways, including acute myeloid leukemia (oas05221), other glycan degradation (oas00511), and ubiquitin mediated proteolysis (oas04120) (Figure 3B).

**Figure 4:**
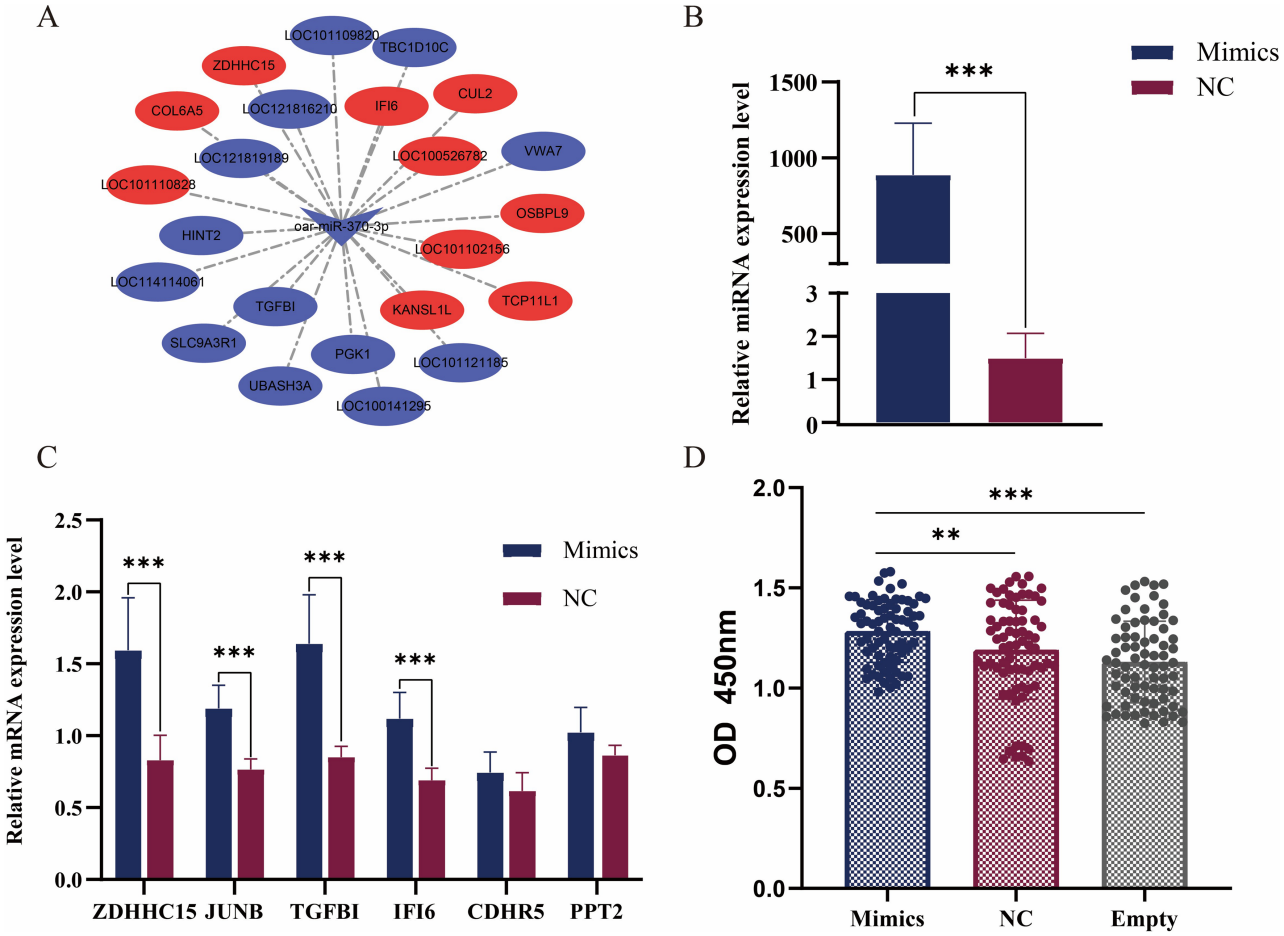
Functional verification of miR-370-3p. **(A)** miR-370-3p and target gene network map. **(B)** Verification of the overexpression effect of miR-370-3p. **(C)** Detection of the expression level of target genes after overexpression of miR-370-3p. **(D)** The effect of miR-370-3p on the activity of sheep fibroblasts. * represent significant difference. ** *p* < 0.01; *** *p* < 0.001.

We combined the transcriptome data of skin tissues of different wool fineness in Ordos fine-wool sheep in the previous stage (9). DE mRNA were determined using the edgeR package, with a *p*-value threshold of ≤ 0.01 to determine statistically significant differences in mRNA expression. By intersecting DE mRNAs with 2,844 target genes, we screened out nine DE miRNAs and 64 differentially expressed target genes. We visualized the DE miRNA-DE mRNA network using Cytoscape. We visualized the DE miRNA-DE mRNA network using Cytoscape. oar-miR-370-3p predicted 23 differentially expressed target genes (Figure 4A).

### 3.4 Functional verification of oar-miR-370-3p

To verify the transfection efficiency of mimics, the results from RT-qPCR detection showed that compared with the control group, the expression level of miR-370-3p in the experimental group was extremely significantly increased (*p* < 0.001) (Figure 4B). The results indicated that miR-370-3p was successfully transfected into sheep fibroblasts. Figure 4C shows that after overexpression of miR-370-3p, the mRNA expression levels of *ZDHHC15*, *JUNB*, *TGFBI*, and *IFI6* increased extremely significantly (*p* < 0.001), while the expression levels of *CDHR5* and *PPT2* remained unchanged. Additionally, Figure 4D indicates that cell activity in the experimental group was significantly higher than that in both the control group (*p* < 0.01) and the empty group (*p* < 0.001). The results indicated that miR-370-3p can promote the growth of sheep fibroblasts.

## 4 Discussion

It is well established that miRNAs exhibit functional specificity, influencing particular tissues and cells, and they are expressed in specific tissues, organs, and developmental stages. We obtained the miRNA expression profile of sheep skin tissue through transcriptome sequencing. The three miRNAs (oar-miR-148a, oar-miR-26a, and oar-miR-143) with the highest average expression levels in the samples were widely expressed in animal tissues. Early reports indicated that miR-148a-3p, miR-26a-5p, and miR-143-3p all had high expression patterns in the mammary glands of dairy cows, goats, mice, and humans (10–12). Notably, miR-148a is expressed in most tissues of normal human bodies and is highly expressed in the liver (13–15). Interestingly, multiple studies have shown that miR-203 is a specific miRNA involved in skin morphogenesis and plays an important role in maintaining skin homeostasis (16–20). In our study, the expression abundance of miR-203 was not detected in sheep skin tissues. The above evidence indicates the specificity of miRNA expression patterns among species. Our research results will provide valuable experimental evidence to enrich the mammalian miRNA database.

We screened 13 known DE miRNAs from the skin tissues of the FG group and CG group of Ordos fine-wool sheep. Among these, miR-127, miR-409-3p, miR-21, miR-200b, and let-7 have been proved to be related to skin healing, immunity, and hair follicle development. The inhibitor of rno-miR-127 can promote the differentiation of hair follicle stem cells (HFSCs) and negatively regulate PODXL2, thereby promoting the repair of skin damage (21). HFSCs are a type of cell with self-renewal and multi-directional differentiation potential located in the protrusion of the outer root sheath. The existence of HFSCs maintains the life cycle of hair follicles and acts as the source of hair growth and regeneration (22). The miR-409-3p also plays an important regulatory role in skin wound healing (23). Tian P et al. discovered that miR-127 blocks the development of melanoma by targeting *DLK1*, providing a new biomarker for the treatment of melanoma. Melanoma is the most common type of skin cancer (24). The miR-21 is associated with longevity and is involved in the differentiation of Langerhans cells (LCs), regulating various immune response processes in the skin (25, 26). In addition, studies have shown that miR-200 is also a potential target for maintaining LC homeostasis (27). miR-200b belongs to the miR-200 family and plays a key role in epithelial-mesenchymal transition (28, 29). let-7 microRNA is a type of miRNA discovered relatively early. A large amount of evidence indicates that let-7 is involved in the regulatory process of the development of multiple organs in animals and is closely related to the occurrence of human diseases (30). Wang Jianmeng et al. found that after melatonin implantation, the expressions of let-7 g and let-7d in the skin of cashmere goats could be down-regulated, thereby affecting the periodic growth of hair follicles (31). Another study showed that let-7 was found as a biomarker of aging in human skin senescent fibroblasts (32).

Three DE miRNAs (oar-miR-495-5p, oar-miR-379-5p, and oar-miR-376e-3p) have been reported in previous studies on the reproductive traits of sheep. Zhang et al. discovered oar-miR-495-5p in the sequencing of hypothalamic tissues in sheep. Multiple miRNAs, including oar-miR-379-5p, oar-miR-148a, and oar-miR-143, may affect the activity of gonadotropin-releasing hormone related to reproductive hormone release and the survival of nerve cells in direct and indirect ways (33). The expression of oar-miR-379-5p was abnormal in the testicular cells of sheep infected with mass dermatological virus (34). The oar-miR-379-5p target *WNT8A* plays a key role in influencing the growth, proliferation and apoptosis of sheep testicular cells (35). Du et al. found that compared with the hypothalamus of sheep, oar-miR-376e-3p was specifically expressed in the adrenal gland (36). In addition, Ma et al. found that miR-376e-3p was differentially expressed significantly in the tail adipose tissues of sheep with different fat-tail types (37). In the studies of ewes with high and low milk production, it was shown that miR-16b was negatively correlated with milk production (38). However, there are few reports on the related studies of oar-miR-323c and oar-miR-544-5p in sheep or skin development and hair follicle growth. The functional studies of these DE miRNAs mainly focus on human tumor diseases, and their specific functions in sheep hair still require further exploration and verification.

We conducted a joint analysis of the DE mRNA identified in the skin tissues of different wool fineness in the previous stage with the DE miRNA target genes in this study (9). Among these, oar-miR-370-3p exhibited the highest number of differentially expressed target genes (Figure 4A). The miR-370-3p is widely expressed in various tissues of sheep and is involved in multiple physiological functions, including embryo implantation and development (39), environmental adaptability (40), reproductive capacity (41), and the replication of vaccine viruses (42). Several proofs indicate that miR-370-3p is related to the development of animal hair follicles. Fu et al. found that miR-370-3p was differentially expressed in the skin tissues of different sheep breeds. It has the ability to target *SMAD4*, thereby inhibiting the proliferation of dermal papilla cells, promote cell apoptosis, and affect the cell cycle (43). Hai et al. reported the differential expression of chi-miR-370-3p in the skin tissues of cashmere goats at different fetal stages. This miRNA inhibited the proliferation of skin cells by regulating the expression of target genes and induced hair follicle morphogenesis (44).

After overexpressing miR-370-3p in sheep skin fibroblasts, miR-370-3p was found to downregulate the expression levels of multiple target genes (Figure 4C), which reflects the diversity and complexity of the regulatory patterns of miRNA in biological processes. Among these, the target gene *TGFBI* promotes the growth of epidermal stem cells and wound healing (45). The *TGFBI* can promote the proliferation of epidermal stem cells and maintains their stemness and cell connectivity through the Wnt/*β*-Catenin pathway (46, 47). The Wnt signaling pathway is essential for hair follicle morphogenesis and hair follicle regeneration. During the process of hair regrowth, the stable expression of β-catenin in the hair germ and protrusions activates the transcription of downstream target genes, thereby promoting the activation and proliferation of hair follicles (48, 49). The TGF-β/BMP signaling pathway determines the growth stage of hair follicles through a negative feedback mechanism (50). The target gene *JUNB* plays an important role in various physiological processes such as cardiovascular development, bone marrow formation, and epidermal tissue homeostasis (51, 52). Wang et al. discovered that *JUNB* is involved in hair follicle differentiation through specific expression during the embryonic period of cashmere goats (53). Another study indicates that *JUNB*, as a target of non-coding RNA, may be involved in the regulation process of cashmere fineness (54).

Mouse experiments have demonstrated that *IFI6* can regulate apoptosis and immune responses, and it has a radiation protection effect on skin cells. Radiation-induced skin damage is a common complication of radiotherapy. The *IFI6* can significantly reduce the radiosensitivity of HaCaT cells (55, 56). Early transcriptome studies have shown that *COL6A5* is associated with the fineness of wool and cashmere (57, 58). KEGG analysis shows that *COL6A5* is annotated in the PI3K-Akt signaling pathway and ECM-receptor interaction. These signaling pathways jointly ensure the development and differentiation of hair follicles and the precise regulation of skin cells. The above evidence indicates that miR-370-3p plays multiple roles in the complex process of hair follicle development and maturation. However, this study still has some limitations: Firstly, the function of miR-370-3p has not yet been verified in *in vivo* animal models; Secondly, we did not explore the interaction relationship between miR-370-3p and other non-coding RNAs (such as lncRNA or circRNA). In addition, miR-370-3p may also regulate other genes in hair follicle development. Future research should focus on verifying its function at the animal level, exploring its regulatory network with other non-coding RNAs, and further clarifying the molecular mechanism by which miR-370-3p is involved in hair follicle development and hair growth.

## 5 Conclusion

We identified the miRNA expression profile in the skin tissue of Ordos fine-wool sheep through transcriptome sequencing. The key DE miRNA, oar-miR-370-3p, was screened in skin tissues with different fiber diameters. Cell experiments have shown that oar-miR-370-3p affects the proliferation of fibroblasts by regulating the expression of multiple target genes. These research results provide valuable data resources for understanding the biology of hair follicle development and for unraveling the genetic basis of wool fineness traits.

## Data Availability

The original contributions presented in the study are included in the article, further inquiries can be directed to the corresponding author. The miRNA expression profiles of 20 sheep skin tissues in this paper have been deposited in the China National Center for Bioinformation (ID: OMIX010993).
